# Characterization of Three Polysaccharide-Based Hydrogels Derived from *Laminaria japonica* and Their Hemostatic Properties

**DOI:** 10.3390/md22040188

**Published:** 2024-04-20

**Authors:** Yang Chen, Jinying Shi, Huamai Qiu, Lijun You, Panqi Xu, Rui Rao, Minqian Wu, Ruohan Jia

**Affiliations:** School of Food Science and Engineering, South China University of Technology, Guangzhou 510640, China

**Keywords:** *Laminaria japonica* polysaccharide, hydrogel, extraction, hemostatic property, swelling property

## Abstract

Three *Laminaria japonica* polysaccharides (LJPs) extracted via water extraction (LJP-W), acid extraction (LJP-A), and enzymatic extraction (LJP-E) were used as raw materials to be cross-linked with chitosan and polyvinyl alcohol to prepare hydrogels. Compared with conventional hydrogel systems, all three types of LJP-based polysaccharide hydrogels exhibited better swelling properties (14 times their original weight) and the absorption ability of simulated body fluid (first 2 h: 6–10%). They also demonstrated better rigidity and mechanical strength. Young’s modulus of LJP-E was 4 times that of the blank. In terms of hemostatic properties, all three polysaccharide hydrogels did not show significant cytotoxic and hemolytic properties. The enzyme- and acid-extracted hydrogels (LJP-Gel-A and LJP-Gel-E) demonstrated better whole-blood coagulant ability compared with the water-extracted hydrogel (LJP-Gel-W), as evidenced by the whole blood coagulation index being half that of LJP-Gel-W. Additionally, the lactate dehydrogenase viabilities of LJP-Gel-A and LJP-Gel-E were significantly higher, at about four and three times those of water extraction, respectively. The above results suggested that LJP-Gel-A and LJP-Gel-E exhibited better blood coagulation capabilities than LJP-Gel-W, due to their enhanced platelet enrichment and adhesion properties. Consequently, these hydrogels are more conducive to promoting coagulation and have good potential for wound hemostasis.

## 1. Introduction

As the largest organ of the human body, the skin serves as the initial defense mechanism of the body by protecting the internal environment, resisting bacterial invasion, and avoiding excessive water loss. However, once the skin is damaged and fails to heal promptly, it can lead to a variety of health problems such as bacterial infections, water and protein loss, and immune system disorders [1,2,3]. When confronted with a mechanical injury, the skin initiates a complex wound healing process that consists of four phases [4]: hemostasis [5], inflammatory response [6], cell proliferation, and tissue remodeling [7]. Among these stages, rapid and effective hemostasis is essential to facilitating the wound-healing process.

Conventional suture hemostasis has some drawbacks, such as slow hemostasis and susceptibility to infection [8]. Conventional hemostatic materials, such as cotton gauze and cotton pads, may adhere to the bleeding tissue, are difficult to remove, and are difficult to apply to irregular and narrow injuries [9]. Although some hemostatic materials, such as zeolite, chitosan, and kaolin, provide good hemostasis, there is a risk of secondary damage to the traumatic tissue [10]. The development of large organic compounds such as proteins and polysaccharides for use as hemostatic materials is currently receiving more and more attention from scientists. Protein-based gelatin sponges may come with problems such as the triggering of allergic reactions [11], high risks of infection, and high costs. However, polysaccharide-based hydrogels [12] might provide new ideas and breakthroughs for the development of hemostatic materials [13,14].

Hydrogel is a biodegradable natural hydrophilic polymer with a thorough three-dimensional mesh structure that can retain a large amount of water without dissolving the cross-linked polymer, showing good biocompatibility, flexibility, and plasticity [15], and has mechanical properties that make it feasible for a wide range of applications in the chemical and biomedical fields [16,17]. Researchers have developed protein/polymer composite hydrogels for slow drug release [18]. *Laminaria japonica* polysaccharide (LJP) has various biological activities such as hypolipidemic, antioxidant, anti-radiation, anti-inflammatory, antibacterial and immune-regulatory activities [19]. The extraction methods of *Laminaria japonica* polysaccharides mainly include hot water extraction [20], acid extraction [20], enzyme extraction [21], etc. The LJPs extracted via different solvents and methods have significant differences in extraction rate, structure, and functional activities [22,23].

In this study, *Laminaria japonica* polysaccharides from *Laminaria japonica* (LJPs) were prepared using three methods: water extraction, acid extraction, and enzymatic digestion. The chemical compositions of the three polysaccharides were analyzed. Then, LJPs were cross-linked with chitosan and polyvinyl alcohol to prepare hydrogels. The hemostatic properties of the three hydrogels will be evaluated via a quantitative analysis of the hemolysis rate, whole-blood absorption capacity, and coagulation capacity, and via platelet quantitative analysis. This study will provide a significant scientific foundation for the innovative application of *Laminaria japonica* polysaccharides and promote the further innovation and development of hemostatic materials.

## 2. Results

### 2.1. Chemical Compositions of Three LJPs

The chemical compositions of the three LJPs are shown in Table 1. The total sugar contents of the LJPs, determined via water extraction, acid extraction and enzyme extraction (LJP-W, LJP-A, and LJP-E), were 85.27 ± 0.53%, 95.12 ± 0.77%, and 95.86 ± 0.72%, respectively. The results showed that the extraction rates of the acid extraction and enzyme extraction (LJP-A and LJP-E) LJPs were higher than that of the water extraction LJP (LJP-W). LJPs show strong water retention and adhesion in hydrogels, which help to form a gel mesh structure, thus promoting a hemostatic effect [24]. The galactose glucuronide contents of the above three LJPs were 25.08 ± 1.78%, 24.87 ± 1.04%, and 26.15 ± 3.62%, respectively. The glucose glucuronide contents were 35.92 ± 2.51%, 41.81 ± 0.58%, and 43.69 ± 3.46%, respectively. The available literature indicates that the content of glucuronic acid in polysaccharide greatly affects its biological activity.

From Table 2, it can be seen that the main components of the LJPs are glucuronic acid, galacturonic acid, fucose, and galactose. In addition, LJP-W also contained xylose, glucose, and arabinose. The contents of the three sugars, galacturonic acid, galactose, and fucose, in LJP-W were similar. The contents of fucose and galactose in LJP-W were higher than those in LJP-A and LJP-E. The contents of glucuronic acid in LJP-A and LJP-E (43.69%, 41.81%) were significantly higher than that in LJP-W (35.92%).

The components of LJP belonging to brown algae polysaccharides mainly include alginate (mainly composed of uronic acid), laminarin (a polysaccharide containing sulfate groups), and fucose (mainly composed of glucose). According to the results of monosaccharide composition, it can be inferred that the main component of LJP is uronic acid, and the contents of uronic acid in polysaccharides extracted via the three methods were 61.00%, 66.68%, and 69.84%. It can be speculated that the content of alginate in LJP is high. As a linear anionic polysaccharide, alginate contains carboxyl groups that can interact with multivalent cations (such as magnesium, calcium, barium, iron, etc.), undergo an almost temperature-independent sol–gel transition under relatively mild conditions, and form hydrogels [25]. In addition, LJP also had a high content of fucose and galactose, while the contents of glucose and xylose were relatively low, indicating that LJP also contained a considerable amount of laminarin, with a low content of fucose fucoidan [20].

### 2.2. Characterization of Three Hydrogels

#### 2.2.1. Swelling Properties

**Figure 1:**
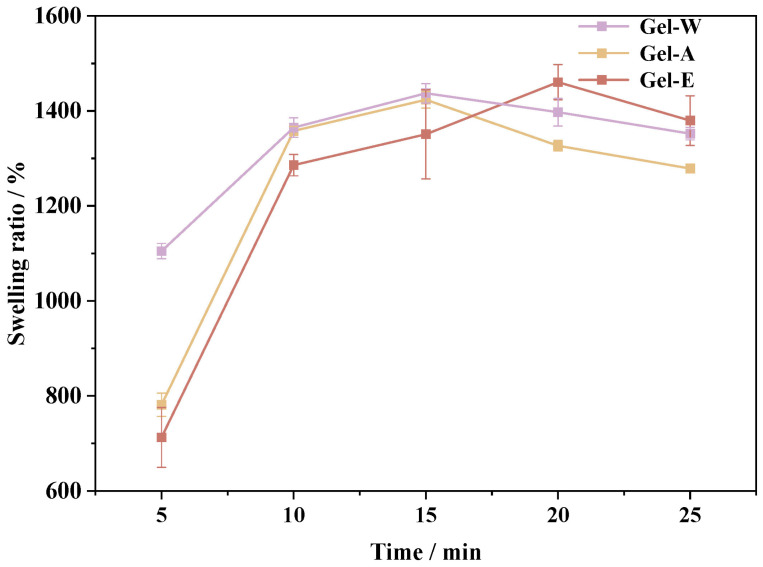
The swelling ratio of three LJP-based hydrogels.

The water absorption capacity of a hydrogel is one of the important factors in maintaining a moist environment on the wound surface [26]. The swelling rate of the LJP-based hydrogels, extracted via the above three methods in distilled water, is shown in Figure 1. In the initial stage of swelling, the first 15 min of swelling, water was quickly absorbed by the three kinds of hydrogels, which achieved an equilibrium swelling rate of more than 85%. Among them, the equilibrium swelling rates of LJP-Gel-W and LJP-Gel-A reached 1437% and 1423%, respectively. However, the swelling equilibrium was reached by LJP-Gel-E after 20 min, with the highest equilibrium swelling rate among the three hydrogels, at 1460%. Compared with the conventional hydrogel system, all three LJP-based hydrogels had better swelling properties [27]. However, as the swelling time increased, the swelling rates of the three hydrogels gradually reduced. On the one hand, in the early stage of swelling, hydrophilic structures of the polysaccharides like -OH and -COOH were rapidly extended, resulting in a large number of water molecules quickly entering the internal cavity structure of the hydrogel and the swelling rate increasing rapidly [28]. On the other hand, with increasing swelling time, the structures not fully cross-linked in the hydrogel were dissolved in water, resulting in a decrease in hydrogel weight.

#### 2.2.2. Mechanical Property Analysis

**Figure 2:**
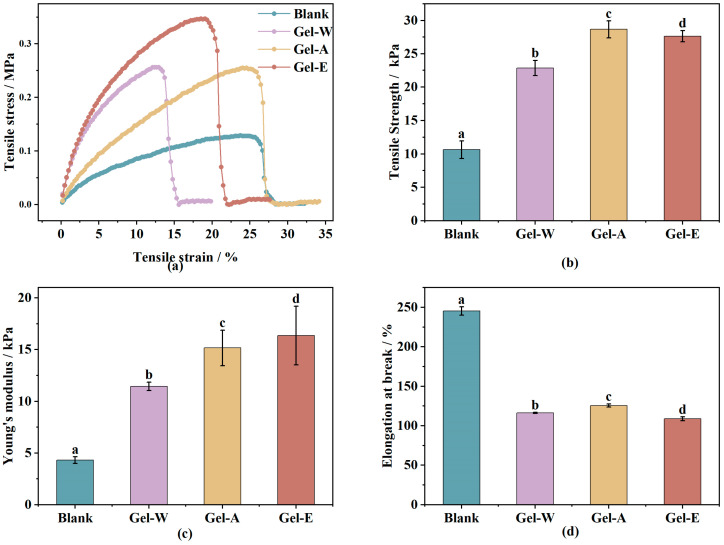
The mechanical properties of three LJP-based hydrogels’ stress–strain curves (**a**), tensile strength (**b**), Young’s modulus (**c**) and elongation at break (**d**). Values with different letters are significantly different (*p* < 0.05).

The excellent mechanical properties enable the hydrogels to maintain their original shape, which plays an important role in their application as hemostatic dressings as well as biomaterials. From the stress–strain curves in Figure 2a, it can be seen that the samples experienced repeated fluctuations and step-type fracture processes. This indicates that the *Laminaria japonica* polysaccharide-based hydrogel is not a brittle material but a soft and ductile material. The hydrogel has a certain ability to maintain its original shape when stretched by an external force, which leads to the fluctuating shape of the stress–strain curves. The tensile strength of the hydrogels is shown in Figure 2b. The tensile strength of the blank sample (chitosan/PVA) was 10.63 ± 1.31 kPa, and when the *Laminaria japonica* polysaccharides obtained from different extraction methods (LJP-W, LJP-A, and LJP-E) were introduced into the hydrogel system, the tensile strength of the hydrogels was enhanced to 22.85 ± 1.14, 28.67 ± 1.26, and 27.63 ± 0.86 kPa. Their Young’s modulus, which is shown in Figure 2c, also increased from 4.31 ± 0.32 to 11.45 ± 0.41, 15.16 ± 1.72, and 16.37 ± 2.84 kPa, respectively. On the contrary, as shown in Figure 2d, the introduction of *Laminaria japonica* polysaccharides decreased the elongation at break of the hydrogels from 245.31 ± 5.32% to 116.29 ± 0.69%, 125.65 ± 2.01%, and 108.74 ± 2.48%, respectively.

The greater the tensile strength, the greater the tensile stress that the hydrogel can withstand and the greater the rigidity of the sample material. Similarly, Young’s modulus is used to characterize the rigidity of the material; the higher the Young’s modulus, the less prone the material is to deformation and the more rigid it is. The higher the growth rate at break, the better the toughness of the material. Therefore, the results of the tensile experiments indicated that the introduction of *Laminaria japonica* polysaccharides into the chitosan/PVA system could transform the material from exhibiting toughness to rigidity and enhance the mechanical strength.

From the hydrogel structural point of view, the low mechanical strength of chitosan/PVA hydrogels can be attributed to the single cross-linking mode and the lack of an energy dissipation mechanism [29]. In a chitosan/PVA hydrogel system, PVA forms a network structure after repeated freezing and thawing, and chitosan does not additionally interact with PVA to increase the cross-linking degree of the hydrogel. Therefore, chitosan/PVA hydrogels have better toughness, but low tensile strength and a low Young’s modulus. When *Laminaria japonica* polysaccharide was introduced, the free hydroxyl and carboxyl groups in *Laminaria japonica* polysaccharide and the amide groups of chitosan formed a rigid network structure with high cross-linking density through interactions, while PVA also formed a soft, low-cross-linking, and dense network structure after repeated freezing and thawing [27]. Thus, the obtained *Laminaria japonica* polysaccharide/chitosan/PVA hydrogel was a double-network hydrogel with an energy-dissipating mechanism. The dual-network hydrogel consists of a rigid first-layer network with high cross-linking density and a soft, low-cross-linking, dense second-layer network [30]. In the dual-network hydrogel, the rigid network acts as a sacrificial bond and the network breaks preferentially, and the breakage leads to energy loss, which improves the mechanical properties of the hydrogel, while the second-layer network (flexible network) provides elasticity to the hydrogel [31]. This dual-network hydrogel can provide a solution to the disadvantages of commercial dressings such as their low mechanical properties and the fact that they are to break after blood absorption, causing great pain to the affected area when changing the dressing.

### 2.3. Absorption Capacity of Simulated Body Fluid (SBF)

**Figure 3:**
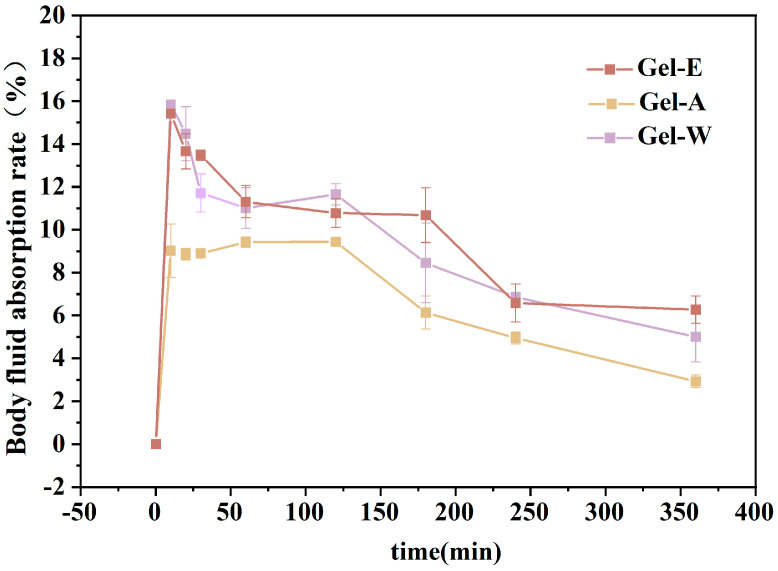
Simulated body fluid absorption of three LJP-based hydrogels.

An ideal hydrogel with hemostatic properties needs to absorb a certain amount of tissue fluid exudate [32], so this study simulated the human body fluid environment. The hydrogel was placed in the simulated body fluid, and the absorptive body fluid properties of the hydrogel were measured. As can be seen in Figure 3, the three kinds of LJP-based hydrogels all had better swelling properties. Among them, the water extraction and enzyme extraction LJPs (LJP-Gel-W, LJP-Gel-E) had better absorptive body fluid properties than that of the acid extraction LJP (LJP-Gel-A), and could maintain 10% water absorption within the first two hours. This might have been because the acid treatment disrupted the glycosidic bonds in the polysaccharide structure and reduced the molecular weight of the *Laminaria japonica* polysaccharides, forming more small-molecule short-chain polysaccharides, which resulted in more sparse three-dimensional network structures of LJP-Gel-A and weaker absorptive performance for body fluids [23,33]. In contrast, LJP-Gel-W and LJP-Gel-E retained a more complete polysaccharide structure, making their hydrogel structures denser and therefore more absorbent for body fluids. Subsequently, with the increase in time, the water absorption gradually decreased but still managed to stay above 0%. This might have been related to the special structure of the polysaccharide-based hydrogel of *Laminaria japonica* [33].

### 2.4. Hemolysis Rate

**Figure 4:**
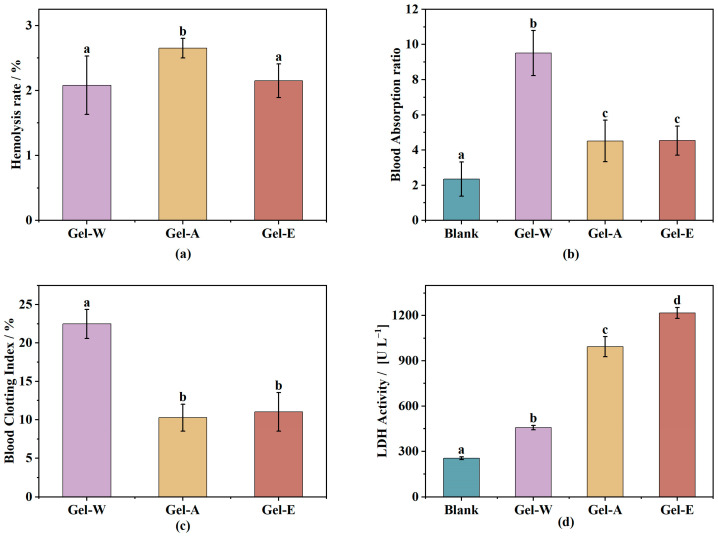
The hemostatic properties of three LJP-based hydrogels’ hemolysis rate (**a**), blood absorption (**b**), blood clotting index (**c**), and LDH activity (**d**). Differences between means with the same exponent letter are not significant at a *p*-level value < 0.05.

Hemolysis is the rupture of red blood cells in the blood and the escape of hemoglobin [34]. In the human body, hemolysis may be caused by various mechanical injuries, the invasion of hemolytic bacteria, antigen–antibody reactions, intrinsic defects in red blood cells, or side effects of certain drugs [35]. The hemolysis rate is an indicator for evaluating the biocompatibility of wound dressings. Usually, a hemolysis rate of less than 5% indicates that the sample can be compatible with blood and will not cause hemolysis [36]. As shown in Figure 4a, the three hydrogel samples were compatible with blood, in which the hemolysis rates of hydrogels prepared, i.e., the water extraction, acid extraction and enzyme extraction LJPs (LJP-Gel-W, LJP-Gel-A, and LJP-Gel-E), were 2.08 ± 0.46%, 2.65 ± 0.15%, and 2.15 ± 0.26%, respectively; none of them showed significant hematotoxicity and could be used in wound dressings for hemostasis.

### 2.5. Absorption of Whole Blood Properties

The results of Figure 4b show that the whole-blood absorption rates of Blank, LJP-Gel-W, LJP-Gel-A, and LJP-Gel-E reached 2.33 ± 1.02, 9.51 ± 1.02, 4.52 ± 1.18, and 4.54 ± 0.82 times the weight of the hydrogel itself, respectively. The blood absorption rates of LJP/chitosan/PVA hydrogels were significantly higher than those of the chitosan/PVA hydrogels, which was mainly due to the dual-network structure of the LJP/chitosan/PVA hydrogels. As mentioned earlier, in a LJP/chitosan/PVA hydrogel, the negatively charged LJP and positively charged chitosan forms a network structure through electrostatic interactions [37]. At the same time, PVA forms a second network structure through repeated freezing and thawing, and thus the resulting LJP/chitosan/PVA hydrogel is a dual-network structure hydrogel with both high swelling properties and high strength. This loose and stable structure facilitates the absorption of blood into the hydrogel [38].

Among the LJP/chitosan/PVA hydrogels, LJP-Gel-W had the highest rate of whole-blood absorption, reaching 9.5 times the original weight. There was no significant difference between the two samples of LJP-Gel-A and LJP-Gel-E (*p* > 0.05), which may have been due to the more dense structure of the two types of gels, LJP-Gel-A and LJP-Gel-E. Considering with the above results, the *Laminaria japonica* polysaccharide-based hydrogels exhibited a higher whole-blood absorption rate and a better procoagulant effect, and could be used for wound hemostasis.

### 2.6. The Whole-Blood Coagulation Index

The whole-blood coagulation index (BCI) is an important index for evaluating the hemostatic performance of biomaterials, and the higher the BCI, the slower the blood coagulation rate and the worse the hemostatic performance [39]. In this study, the absorbance value of the unabsorbed blood of the gel sample was measured to be compared with that of the control group. The BCI values are shown in Figure 4c. The results in the figure show that the BCI values of hydrogels prepared, i.e., the water extraction, acid extraction, and enzyme extraction LJPs (LJP-Gel-W, LJP-Gel-A, and LJP-Gel-E), were 22.4 ± 1.89%, 10.27 ± 1.75%, and 11.03 ± 2.50%, respectively. The BCI values of hydrogels prepared via acid extraction and enzyme extraction (LJP-Gel-A and LJP-Gel-E) were significantly lower than that of the water extraction LJP (LJP-Gel-W), and had better procoagulant properties.

### 2.7. Quantitative Platelet Analysis

The hemostatic process consists of three main stages: vasoconstriction, platelet thrombosis, and blood coagulation. Among them, platelet aggregation and adhesion are important factors affecting the hemostatic effect of biomaterials. Under normal conditions, platelets in human blood are in a resting state. When an individual is injured and bleeding, platelets are immediately activated, aggregating and adhering to the wound to form a platelet thrombus [40].

Lactate dehydrogenase (LDH) plays an important role in platelet generation, and platelet aggregation and adhesion are important factors affecting the hemostatic effect of biomaterials. In in vitro experiments, the higher the activity of LDH, the more platelets can be adsorbed, which is more conducive to in vitro wound hemostasis. As shown in Figure 4d, the LDH viabilities of Blank, LJP-Gel-W, LJP-Gel-A, and LJP-Gel-E were 255.65 ± 9.42, 457.53 ± 14.34, 992.64 ± 66.65, and 1216.43 ± 36.48 U/L, respectively, which shows that LJP-Gel-A and LJP-Gel-E had the highest LDH activities, followed by LJP-Gel-W, and the activities of all were significantly higher than that of the Blank control. This indicates that compared with the Blank, the *Laminaria japonica* polysaccharide-based hydrogel was able to enrich and adhere to a larger number of platelets, which was more conducive to the hemostatic activity of the hydrogel. Meanwhile, the LDH activities of LJP-Gel-E and LJP-Gel-A were significantly higher than that of LJP-Gel-W (4 and 3 times higher, respectively). On one hand, the loose and porous structure of the *Laminaria japonica* polysaccharide-based hydrogel showed that it could hold more water, and the internal network had a high cross-linking density and a stable structure that was not easy to be destroyed. Therefore, when it came into contact with blood, LJP-Gel-E and LJP-Gel-A rapidly absorbed the water in the blood, concentrated the blood, and promoted the concentration and aggregation of platelets. On the other hand, when the material had an electric charge on its surface, it could adsorb platelets through an electrostatic effect and promote the aggregation of platelets to achieve the effect of hemostasis.

### 2.8. Cell Cytotoxicity

**Figure 5:**
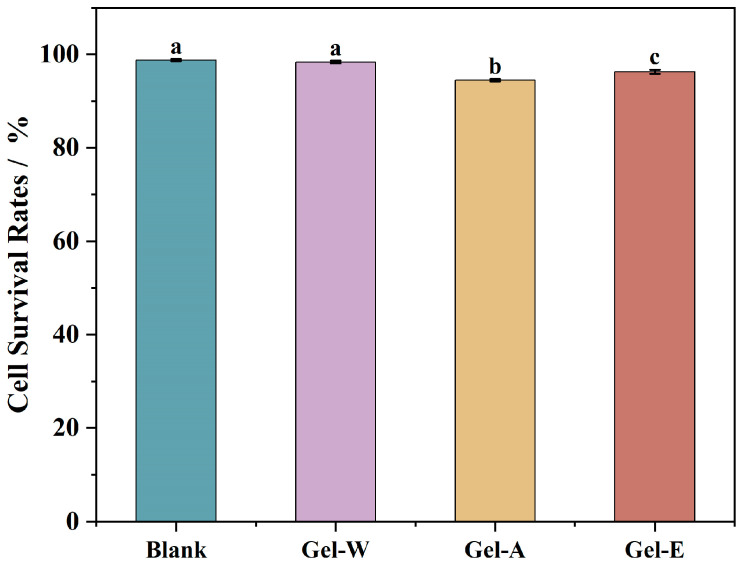
The cell cytotoxicity of the three LJP-based hydrogels. Values with different letters are significantly different (*p* < 0.05).

HaCaT cells were selected to evaluate the cytotoxicity of the LJP-based hydrogels. HaCaT cell is a type of human epidermal immortalized cell that has high proliferative capacity and epidermal cell characteristics [41]. The toxicity of three LJP-based hydrogels to HaCaT cells was evaluated by directly putting them into contact with the cells for 24 h, and their cell survival rates were determined. As shown in Figure 5, the results showed that after exposure, the three hydrogel samples had very limited effects on HaCaT cells, with cell survival rates of 98.34 ± 0.7%, 94.46 ± 0.22%, and 96.27 ± 0.43%. This indicates that the LJP-based hydrogels did not show significant cytotoxicity to HaCaT cells and can be considered safe hemostatic materials.

## 3. Materials and Methods

### 3.1. Materials and Chemicals

*Laminaria japonica* was obtained from Fuzhou, China. Chitosan (degree of deacetylation = 90%) was purchased from Shanghai Yuanye Bio-Technology Co., Ltd. (Shanghai, China). Citric acid (analytical reagent grade) was purchased from Maclin Biochemical Reagent Co., Ltd. (Shanghai, China). Fresh sheep blood was purchased from Guangzhou, China. The LDH kit was purchased from Nanjing Jiancheng Bio-Technology Co., Ltd. (Nanjing, China). Human immortalized keratinocytes (HaCaT cells) were obtained from Cell Resource Center, Shanghai Institute of Biological Sciences (Shanghai, China).

### 3.2. Extraction of LJPs via Different Methods

#### 3.2.1. Pre-Treatment of *Laminaria japonica*

The *Laminaria japonica* was cleaned with tap water, dried in an air oven at 60 °C for 24 h before being crushed using a common pulverizer and passed through a 40-mesh sieve, and then processed using an ultra-micro pulverizer for 5 min. Finally, 95% ethanol was added at a ratio of 1:4 (*w*/*v*) to reflux for 3 h for defatting and decolorization, and then the ethanol was discarded [42]. *Laminaria japonica* superfine powder was obtained via drying at 50 °C.

#### 3.2.2. LJP Extraction via Water Extraction

A certain amount of pretreated *Laminaria japonica* powder was weighed, and deionized water was added at a ratio of 1:50 (*w*/*v*) and extracted at 100 °C for 4 h. After the extraction, the solution was centrifuged at 8000 r/min for 15 min, and the supernatant was collected and concentrated to 1/4 of the original volume via vacuum spinning; anhydrous ethanol was added to the solution to a concentration of 80%, and the solution was placed in a refrigerator at 4 °C for 12 h. The sample was centrifuged at 8000× *g* r/min for 10 min, the precipitate was collected, the ethanol was allowed to evaporate, water was added to re-dissolve it, and then vacuum lyophilization was performed to obtain the hot water-extracted polysaccharide (the sample named LJP-W).

#### 3.2.3. LJP Extraction via Acid Extraction

A certain amount of pretreated *Laminaria japonica* powder was weighed, and citric acid (pH = 2) was added at a material–liquid ratio of 1:50 (*w*/*v*); extraction was carried out at 100 °C for 4 h, and the pH was adjusted to neutral with a 2% sodium carbonate solution. After the extraction was completed, the extract was centrifuged, concentrated, alcoholically precipitated, re-solubilized, and lyophilized in the same way as the water extract was. The *Laminaria japonica* polysaccharide extracted via acid extraction was obtained (the sample named LJP-A).

#### 3.2.4. LJP Extraction via Enzymatic Extraction

After weighing a certain amount of pretreated *Laminaria japonica* powder, deionized water was added at a ratio of 1:30 (*w*/*v*), and cellulase was added into the water solution at 0.1% of the final solution, stirred well, and then placed in a 50 °C-water bath for 4 h. After the enzyme was inactivated, deionized water was added again to make the final material–liquid ratio 1:50 (*w*/*v*), and then it was placed in a water bath at 100 °C to extract the polysaccharides for 4 h. The subsequent steps were conducted in the same way as they were in the water extraction method, and the enzyme-assisted water polysaccharide was obtained (the sample named LJP-E).

### 3.3. Chemical Compositions of LJPs

#### 3.3.1. Basic Compositions

The total carbohydrate contents of the three LJPs were determined via the phenol-sulfuric acid method, using fructose solution as the standard. The glycuronic acid content was determined via the sulfuric acid-carbazole method, using galacturonic acid and glucuronic acid as standards. The protein content was determined via the colorimetric method using the colorimetric method of Coomassie Brilliant Blue, with bovine serum protein solution as the standard.

#### 3.3.2. Monosaccharide Composition

The pretreatment of the samples involved dissolving the polysaccharide samples in 2 M trifluoroacetic acid (TFA, 5 mL) and hydrolyzing them for 4 h at 105 °C. Next, 5 mL of methanol was added to a rotary evaporator (Hei-VAP Value Digital, Heidolf instruments, Ltd., Schwabach, Bavaria, Germany) set to evaporate leftover TFA at 60 °C while also evaporating the solvent. The processes were repeated 5 times. After dissolving the residues in distilled water to a pH 6.0–8.0 concentration of 0.1–0.5 mg/mL, a 0.22 μm filter was used to filter the mixture.

Ion chromatography (ISC-3000, Thermo Scientific Dionex Ltd., Sunnyvale, CA, USA) fitted with a CarboPacTM PA20 separation column (3 × 150 mm) was used to determine the monosaccharide compositions of the three LJPs. The column temperature was set at 30 °C, and the flow rate was set at 5 mL/min. For the purpose of pre-operating the instrument, 90% A phase (ultrapure water) combined with 10% D phase (20 mM sodium hydroxide) was eluted for 18 min after 100% B phase (200 mM sodium hydroxide) was eluted for 2 min. After sampling, 90% A phase mixed with 10% D phase was eluted for 15 min, then 70% A phase, 20% C phase (500 mM sodium acetate), and 10% D phase were eluted for 15 min [43].

### 3.4. Preparation of LJP/Chitosan/PVA Composite Hydrogels

The LJP-based hydrogel was prepared with 1% chitosan solution (pH adjusted to approximately 3.0), 0.5% polysaccharide solution, and 5% PVA solution (previously dissolved at 90 °C). The polysaccharide solution (10 mL) was placed in a conical flask with the same volume of chitosan solution being slowly added while stirring, followed by stirring for 5 min. PVA solution (10 mL) was placed in a conical flask and heated in a water bath to 50 °C, after which the crude polysaccharide–chitosan solution was slowly added while stirring. After being stirred evenly, the solution was poured into the mold, then frozen in a −20 °C refrigerator for 4 h, melted at room temperature for 1.5 h, and subjected to freeze–thaw cycles 4 times. After the last melting stage, the hydrogel was washed three times with distilled water. The samples were named LJP-Gel-W, LJP-Gel-A, and LJP-Gel-E, respectively.

### 3.5. Characterization of Hydrogels

#### 3.5.1. Swelling Property Analysis

The dried hydrogel (20 mg) was weighed, soaked in 40–50 mL of deionized water at room temperature in 5 min intervals, and clamped out with tweezers, while excess water was sucked from the surface with filter paper, and the weight of the hydrogel was recorded. Each sample was treated in three parallel runs. The weight of hydrogel at t min was expressed as W_t_, and the dry weight of hydrogel was expressed as W_0_.

(1) $$\mathrm{Swelling}\;\mathrm{ratio}=\frac{W_{t}-W_{0}}{W_{0}}$$

#### 3.5.2. Mechanical Property Determination

The tensile properties of the hydrogel were determined by using a universal material tester. The hydrogel was cut into 10 × 40 mm strips with a thickness of about 2–4 mm. The stretching rate was set to 50 mm/min, the intercept was 40 mm, and each sample was treated in three parallel runs.

Stress (stress/MPa) was taken as the ordinate and strain (strain/%) as the abscissa. The Blank represents a hydrogel sample made with distilled water instead of the polysaccharide solution. The mechanical properties of hydrogels could be characterized by their tensile strength, elongation at break, and Young’s modulus accordingly.

① Tensile strength: Tensile strength is expressed as the maximum breaking force per initial cross-sectional area of the hydrogel.

(2) $$TS=\frac{F_{max}}{A}$$

where F_max_ is represented as the maximum stress that just breaks the hydrogel, and A is represented as the initial cross-sectional area of the sample hydrogel.

② Elongation at break: The elongation at break is expressed as the percentage of the hydrogel length to the original length at fracture.

(3) $$E=\frac{L}{L_{0}}\times 100\%$$

where L is represented as the maximum length of the hydrogel, and L_0_ is represented as the initial length of the hydrogel.

③ Young’s modulus: Young’s modulus, representing the elastic modulus, is the physical quantity characterizing the tensile or compression resistance of the material within the elastic limit. A formula holds for linear elastic materials:

σ = E ε (4)

where σ is the positive stress, ε is the positive strain, and E is the elastic modulus, which is a constant related to the material itself. According to the stress–strain curve, the linear part is selected for calculation. The data points with 30% and 90% are selected to obtain Young’s modulus:

(5) $$E=\frac{\sigma_{90\%}-\sigma_{30\%}}{\varepsilon_{90\%}-\varepsilon_{30\%}}$$

#### 3.5.3. Rate of Absorption of Simulated Body Fluid

The simulated body fluid was prepared as follows: 7.996 g of NaCl, 0.35 g of NaHCO_3_, 0.244 g of KCl, 0.228 g of K_2_HPO_4_-3H_2_O, 0.305 g of MgCl_2_-6H_2_O, 0.278 g of CaCl_2_, 0.071 g of Na_2_SO_4_, 6.057 g of NH_2_C(CH_2_OH)_3_ and 40 mL of HCl (1 mol/L) were dissolved in 1 L of deionized water, sterilized, and stored at 4 °C.

The moist hydrogels were cut into 8 × 8 × 8 mm cubes, submerged in 40–50 mL of simulated body fluid, and weighed after 0, 10, 20, 30, 60, 120, 180, 240, and 360 min.

Before weighing, the hydrogels were fished out and placed on a sieve to dry for 30 min until no more water dripped down. Three parallels runs were conducted for each sample, and water absorption was calculated.

(6) $$\mathrm{SBF}\;\mathrm{absorption}\;\mathrm{ratio}\;(\%)=\frac{W_{t}-W_{0}}{W_{0}}\times 100\%\;$$

where *W_t_* represents the weight of the hydrogel at time *t*, while *W*_0_ represents the original weight of the hydrogel.

### 3.6. Hemostatic Properties Analysis

#### 3.6.1. Hemolysis Rate

About 5 mg of the hydrogel sample was weighed into a 10 mL centrifuge tube, and then 5 mL of anticoagulated sheep’s blood diluted with PBS (the ratio of anticoagulated sheep’s blood to PBS buffer was 4:5) was added. At the same time, PBS + anticoagulated sheep’s blood was used as negative control, *A*_n_, and distilled water + anticoagulated sheep’s blood was used as positive control, *A*_p_. The above samples were placed in a water bath at 37 °C for 1 h, followed by undergoing centrifugation at 3000× *g* rpm for 5 min; the supernatant was taken to measure the absorbance value at 545 nm, and the hemolysis rate was calculated.

(7) $$Hemolysis\;rate\left(\%\right)=\frac{A_{sample}-A_{n}}{A_{p}-A_{n}}\times 100\%$$

#### 3.6.2. Absorption of Whole Blood Properties

A dry hydrogel sample of about 20 mg was weighed and placed in an appropriate amount of anticoagulant sheep’s blood for 1 min, after which the hydrogel sample was placed on a filter paper, which was placed on a funnel, anticoagulant sheep’s blood was added to the hydrogel sample drop by drop at a rate of 1 mL/min until the first drop of blood fell from the funnel, and the weights of hydrogels before and after absorption of anticoagulant sheep’s blood were weighed and calculate.

(8) $$\mathrm{Absorption}\;\mathrm{rate}\;(\%)=\frac{W_{t}-W_{0}}{W_{0}}\times 100\%$$

where *W_t_* is the weight of the hydrogel at termination and *W*_0_ is the weight of the hydrogel at the beginning.

#### 3.6.3. Blood Coagulation Index (BCI)

The lyophilized hydrogel samples were cut into cylinders with a base area of 2 cm^2^ and a height of 3–5 mm, placed in a 12-well plate, and preheated at 37 °C for 5 min. Then, 100 μL of anticoagulated sheep’s blood activated with 0.1 M CaCl_2_ solution was rapidly dripped onto the surface of each piece of hydrogel (0.1 M CaCl_2_ solution: anticoagulated sheep’s blood in the ratio of 1:10 by volume), and the above samples were warmed in a 37 °C temperature bath for 1 h. Subsequently, 5 mL of deionized water was added to each well, and the mixture was shaken on a pendulum bed at 50 rpm for 10 min. The samples were warm-bathed at 37 °C for 1 h. Afterward, 5 mL of deionized water was added to each well, the samples were shaken at 50 rpm for 10 min in a swinging bed to wash away the uncoagulated blood, and then the liquid in the 12-well plate was taken to determine the absorbance value at 540 nm and calculate the blood clotting index (BCI):

(9) $$BCI\left(\%\right)=\frac{A_{sample}}{A_{blank}}\times 100\%$$

#### 3.6.4. Quantitative Platelet Analysis

Lyophilized hydrogels were cut into cylinders with a base area of 2 cm^2^ and a height of 3–5 mm and placed in a 12-well plate. Anticoagulated sheep’s blood was centrifuged at 3000× *g* rpm for 5 min, the supernatant was collected as a platelet-enriched solution, 1 mL of the platelet-enriched solution was rapidly dripped onto the surface of each hydrogel, and the blood that was not adsorbed on the plate was aspirated in a warm bath at 37 °C for 1 h. The plate was washed three times using PBS, and 1 mL of 1% TritonX-100 solution was dripped onto the surface of each hydrogel. After 1 mL of 1% TritonX-100 solution was dropped onto the surface of each hydrogel, the supernatant was taken after 1 h of treatment at room temperature, and the lactate dehydrogenase (LDH) content was determined in accordance with the method of the LDH kit.

### 3.7. Cell Cytotoxicity

#### 3.7.1. Cell Culture

Human keratinocyte (HaCaT) cells were cultured at 37 °C in a 5% CO_2_ environment (a 100 μL phosphate buffer was added to the edge (PBS) to prevent the edge effect) using minimum essential medium (MEM) supplemented with 10% FBS and 1% penicillin–streptomycin antibodies. Every two days, the medium had to be replaced, and the cells’ state of growth was promptly monitored.

#### 3.7.2. Pretreatments of LJP and LJP-Gels

After being formed into the appropriate sizes, lyophilized hydrogels, including LJP-Gel-W, LJP-Gel-A, LJP-Gel-E, and Blank, were pre-swelled for 30 min using phosphate-buffered solution. The expanded hydrogels were then sterilized for 30 min by immersing them in 75% ethanol. The hydrogels were then immersed in PBS to eliminate any remaining ethanol after the ethanol was initially eliminated. Every 30 min, the PBS was replaced until all traces of ethyl alcohol were eliminated. After being dissolved in MEM, the LJPs were filtered using a 0.22 μm filter. The concentrations of the LJP solution were diluted to match those of the LJP-based hydrogel systems.

#### 3.7.3. Cell Cytotoxicity in HaCaT

HaCaT cells were seeded at a density of 10^4^/well in 96-well plates. There was a blank control group and a control group on each 96-well plate. The blank group consisted of wells devoid of cells, whereas the control group consisted of wells containing cells without sample treatment. LJP with different methods (LJP- W, LJP-A, LJP-E, and Blank) and pre-sterilized LJP-gels (LJP-Gel-W, LJP-Gel-A, LJP-Gel-E, and Blank) were added many times once HaCaT cells had multiplied to 80%–90% of the well area. Following a 24 h treatment period, the MTT kit was used at 570 nm in conjunction with a microplate reader (Spectra Max 190, Molecular Devices, Sunnyvale, CA, USA) to evaluate the cytotoxicity of the samples.

## 4. Conclusions

In this study, laminaria japonica polysaccharides (LJPs) were extracted from laminaria japonica via three different extraction methods, named LJP-W, LJP-A, and LJP-E. Then, a series of hydrogels were prepared together with chitosan and polyvinyl alcohol to explore the potential of their application as hemostatic materials for wounds. These hydrogels showed better properties in terms of swelling performance, mechanical strength, and biocompatibility. In particular, the hydrogels prepared via enzymatic and acid extraction methods (LJP-Gel-A and LJP-Gel-E) showed good hemostatic properties. Their whole-blood coagulation capacity was markedly enhanced compared with that of the hydrogel derived from water extraction (LJP-Gel-W), as evidenced by a BCI that was only half that of LJP-Gel-W. Furthermore, LJP-Gel-A and LJP-Gel-E demonstrated higher LDH activities than that of the LJP-Gel-W (four and three times higher, respectively). These findings indicate that all three LJP-prepared hydrogels have the potential to improve wound hemostasis. Specifically, LJP-Gel-A and LJP-Gel-E showed greater efficacy in platelet enrichment and adhesion, which significantly enhanced the coagulation effect of the hydrogels. Consequently, the laminaria japonica-based polysaccharide hydrogels prepared through acid and enzyme extraction provide a more effective solution for wound hemostasis and have broad application prospects in the development of trauma first aid and clinical hemostatic materials.

## Figures and Tables

**Table 1:** Chemical composition of three LJPs.

Sample	Total Carbohydrate Content (%)	Galacturonic Acid Content (%)	Glucuronic Acid Content (%)	Protein Content (%)
LJP-W	85.27 ± 0.53	25.08 ± 1.78	35.92 ± 2.51	2.22 ± 0.35
LJP-A	95.12 ± 0.77	24.87 ± 1.04	41.81 ± 0.58	2.14 ± 0.12
LJP-E	95.86 ± 0.72	26.15 ± 3.62	43.69 ± 3.46	1.41 ± 0.93

**Table 2:** Monosaccharide composition of three LJPs.

Molar Percentage (%)	LJP-W	LJP-A	LJP-E
fucose	22.72	17.12	11.07
arabinose	1.82	1.21	0.87
galactose	13.28	9.95	7.11
glucose	1.10	5.02	8.40
galacturonic acid	25.08	24.87	26.15
glucuronic acid	35.92	41.81	43.69
xylose	0.08	0.02	2.70

## Data Availability

Data are available in a publicly accessible repository.
